# A Case of Bilateral Anesthesia Mumps after Cesarean Section under Spinal Anesthesia: A Rare Case and Literature Review

**DOI:** 10.1155/2022/5004358

**Published:** 2022-10-04

**Authors:** Narges Montazeri, Mohammad Hassan Darabi, Kamran Hessami, Ramin Shekouhi, Maryam Sohooli, Tahereh Shamsi, Fateme Bagheri, Mohammad Amin Shahrbaf

**Affiliations:** ^1^Maternal-Fetal Medicine Research Center, Shiraz University of Medical Sciences, Shiraz, Iran; ^2^Department of Obstetrics and Gynecology, Baylor College of Medicine, Houston, TX, USA; ^3^Colorectal Research Center, Department of Surgery, Shiraz University of Medical Sciences, Shiraz, Iran; ^4^School of Medicine, Shiraz University of Medical Sciences, Shiraz, Iran; ^5^Faculty of Medicine, Shahid Beheshti University of Medical Sciences, Tehran, Iran

## Abstract

A 30-year-old pregnant female presented to the emergency department with labor pain in her 39th week of pregnancy. Cesarean section under spinal anesthesia was the chosen route of delivery for this patient due to previous cesarean section in her first pregnancy. The delivery itself was uneventful but seven hours after the surgery, bilateral swelling of parotid glands were noted. Rehydration therapy and a single dose of hydrocortisone (100 mg IV route) were administered, and swellings were completely resolved on postoperative day 2. This is one of the rare cases of anesthesia mumps after spinal anesthesia, and we think rehydration therapy and the single dose corticosteroid may prove useful in these cases.

## 1. Introduction

Acute temporary swelling of the salivary glands, especially parotid glands, termed as “anesthesia mumps” usually occurs during or after surgery and is one of the rare complications of general anesthesia. [1] While majority of reported cases are surgery related, it should be noted that this disease is not exclusive to operation and recovery rooms as it may arise from bedridden Intensive Care Unit patients, too [2]. It often presents as a painless unilateral parotid enlargement that subsides within few days without further sequelae [3]. Scarcely, respiratory distress occurs which requires immediate intervention aiming at securing airway. [4, 5]

In this study, we report a rare case of bilateral postoperative sialadenitis in a young female who underwent caesarian section (C/S) after the spinal anesthesia. To the best of our knowledge, this is one of the few cases of anesthesia mumps occurring after spinal anesthesia. Another unique aspect of this case is the fact that symptoms resolved rather quickly, with the left side enlargement disappearing in ten hours and the right side resolving in less than 24 hours.

## 2. Case Presentation

### 2.1. Present Illness

A 30-year-old female, Gravid 2 Para 1 Live 1, was scheduled for cesarean section (C/S) at gestational age of 39 weeks and 5 days due to a history of previous cesarean section. The patient was a known case of epilepsy which was controlled by Lamotrigine during pregnancy. Her prior obstetrical history included a C/S for delivery of a term newborn due to severe preeclampsia, while the current pregnancy had been uncomplicated. Pelvic examination revealed a 2 centimeters cervical dilation as the fetus presentation was vertex with a longitudinal lie. Membranes were intact, and there were no signs of leakage. All other physical examination findings and routine laboratory tests were found to be normal. The ultrasound studies of the current gestation were normal throughout pregnancy.

### 2.2. Operative Data

After consulting with neurology service 1600 mg of sodium valproate was administered for the patient before performing routine anesthesia monitoring and supportive O_2_ therapy. Induction of spinal anesthesia was performed by injection of 8 milliliters of Marcaine 0.5% and 10 micrograms of fentanyl, through a 12.5 cm, 27-gauge spinal needle in sitting position at the lumbar level of L_3_-L_4_ interspace. After confirmation of the anesthesia, C/S was performed by a Pfannenstiel incision. The surgical procedure was uneventful, and five minutes after the incision, a healthy male newborn with 2750 grams of weight and 1- and 5-minute Apgar score of 10 was born. After delivery, 4 mg of intravenous (IV) ondansetron was administrated for its antiemetic effect.

### 2.3. Follow-Up

**Figure 1:**
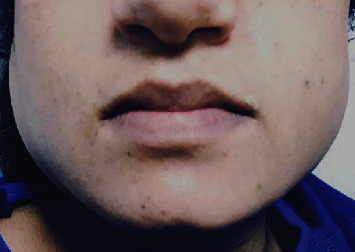
Front face view of the patient at 7 hours after surgery.

The vital signs of patient were normal after the operation, and recovery was uneventful. The patient was transferred to obstetric ward and got out of bed three hours after the surgery. Seven hours after operation, the patient complained from “bilateral facial swelling” (Figure 1).

**Figure 2:**
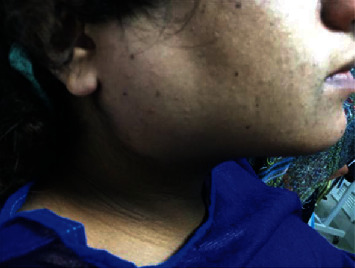
Lateral face view of the patient at 7 hours after surgery.

Physical examination showed bilateral enlargement of parotid regions expanding down to the mandibular angle (Figure 2). There was no pain, redness, or any other sign and symptoms of inflammation. Vital signs including respiratory rate were well within normal values and patient did not complain from dyspnea. Intraoral examination showed patency of Steensen's duct, and no purulent discharge could be seen or elicited by massaging parotid glands. Routine laboratory data including Complete Blood Count were normal with a leucocyte count of 8400/microliter (Table 1). After consultation with Ear Nose Throat (ENT) service, a diagnosis of acute postoperative sialadenitis or anesthesia mumps was established. The patient was hydrated by 1000 cc of normal saline and received one dose of 100 mg IV hydrocortisone afterwards with no antibiotic therapy. The patient was observed every 2 hours, and there was no development of purulent discharge and the left sided swelling healed completely by 10 hours and in postoperative day (POD) 2 the right sided swelling also diminished, although a rise in leucocyte count was observed. On POD 3, patient's general condition, respiration, and the ability to swallow were all normal and she was discharged without any complications.

POD 1: postoperative day 1; POD 2: postoperative day 2.

## 3. Discussion and Literature Review

Acute postoperative sialadenitis was first described by Munde in 1878 after an oophorectomy surgery [6]. It is a noninfectious process with an unknown etiology. However, some etiologies have been proposed throughout the years as more reports surfaced about this benign self-limiting enlargement of parotid glands. These included factors such as dehydration, hyperflexion of neck during surgery, Stensen duct obstruction, prolonged general anesthesia, venous congestion of head and neck, overreactive parasympathetic tone, and adverse drug reactions like succinylcholine-induced profuse saliva production [1, 4, 7, 8]. Anesthesia mumps clearly have a tendency to present as a unilateral swelling after general anesthesia [1]. This can be explained by the potentially significant role of patient positioning in prolonged surgeries under general anesthesia, as these cases usually develop a unilateral enlargement possibly due to an obstruction of Stensen duct. This was the case in a 3-year-old patient reported by Mutaf and Büyükgüral [9]

Our case developed anesthesia mumps after spinal anesthesia. All of the previous cases of spinal anesthesia were females that developed bilateral swellings just like our case did. [10–13] Our patient had no obvious predisposing factor and review of medications proved fruitless, but we can assume that dehydration has played a role in the development of anesthesia mumps [13] since pregnant women with labor pain tend to forget sufficient water intake. For a comparison between our case and previous cases of anesthesia mumps after spinal anesthesia refer to Table 2.

Postoperative sialadenitis is generally considered a self-limited benign disease that will be healed within hours to days in the affected individuals [9]. However, some therapies including rehydration and administrating anti-inflammatory medications such as nonsteroidal anti-inflammatory drugs (NSAIDs) or corticosteroids can be helpful for the treatment of this complication [14]. Our patient was hydrated and received a single dose of hydrocortisone, and the results were more promising than what we originally thought, as the patient healed completely in less than 24 hours. We can assume rehydration therapy as a proper first step in management of this disorder. Nevertheless, the definite therapy for anesthesia mumps remains to be found. Protective measures including hydration before surgery, optimal positioning of the patient in operation room, and choosing the best method of anesthesia can be effective for preventing this complication.

All in all, acute unilateral or bilateral swelling of the parotid gland after anesthesia, particularly of spinal type, is a very rare occurrence. Physicians should be familiar with the transient and benign nature of this anesthesia-related complication which can present itself even after local anesthesia. They should be also prepared for very rare urgent cases that require immediate intervention for securing the airway. According to our case and the review of literature, we can conclude that rehydration therapy with crystalloids such as normal saline is the best first step in management of this disease, although more studies on this matter are required.

## Figures and Tables

**Table 1:** Laboratory findings of the patient.

Variable	POD 1	POD 2
White cell count (per *μ*L)	8400	11900
Lymphocyte count (%)	21.90	20
Neutrophil count (%)	71.8	73
Platelet count (per *μ*L)	230000	180000
Hemoglobin (gr/dL)	10.7	9.3
Red blood cell count × 10^6^ (per *μ*L)	4.93	4
Hematocrit (%)	35.2	27.4
Mean cell volume (FL)	71.4	68.5
Mean corpuscular hemoglobin (Pg)	21.7	23.25
Mean corpuscular hemoglobin concentration (gr/dL)	30.4	33.94
Prothrombin time, control (sec)	12.5	
Prothrombin time, patient (sec)	11.9	
International normalized ratio	1	
Partial thromboplastin time, control (sec)	33	
Partial thromboplastin time, patient (sec)	32	
Blood sugar (mg/dL)	82	
Blood urea nitrogen (mg/dL)	6	
Creatinine (mg/dL)	0.7	
Sodium (mMOL/L)	137	
Potassium (mMOL/L)	3.6	
Blood group & rhesus	A+ positive	

**Table 2:** Published cases of anesthesia mumps following spinal anesthesia in the English literature.

Case	Year	Age	Sex	Symptoms	Surgical procedure	Type of anesthesia	Anesthetic medication	Respiratory distress	Treatment	Complications
1 (11)	2009	89	F	Bilateral painless submandibular gland enlargement	Right partial hip arthroplasty	Combined spinal–epidural anesthesia	15 mg of 0.5% heavy bupivacaine	No	Supportive	Full recovery (5 days)
2 (10)	2013	37	F	Painless bilateral parotid swelling	Breast surgery and abdominoplasty	Thoracic block between T2 and T3 intervertebral spaces, and lumbar epidural block between L1 and L2	13 mL of 0.5% bupivacaine and 50 lg of fentanyl	No	Supportive (IV fluid hydration)	Full recovery (12 hours)
3 (10)	2013	45	F	Painless bilateral parotid swelling	Subglandular breast implantation, and body contouring(liposuction)	Thoracic block between T4 and T5 intervertebral spaces, and lumbar epidural block between L1 and L2	10 mL of 0.5% Marcaine and 100 lg of fentanyl	No	Supportive (IV fluid hydration)	Full recovery (36 hours)
4 (10)	2013	30	F	Painless bilateral parotid swelling	Subglandular breast implantation, and liposuction	Thoracic block between T5 and T6 intervertebral spaces, and lumbar epidural block between L1 and L2	10 mL of 1% lidocaine and 100 lg of fentanyl	No	Dexamethasone injection, and IV fluid hydration	Full recovery (48 hours)
5 (12)	2017	38	F	Mild respiratory distress, and bilateral enlarged parotid gland	Elective cesarean section	Spinal anesthesia	Not mentioned	Yes	Antihistamines, and anti-inflammatory agents, supportive therapy	Full recovery (10 hours)
6 (13)	2021	31	F	Painless swelling in both parotid glands	Elective cesarean section	Spinal anesthesia between L3-L4 intervertebral spaces	12 mg bupivacaine, 100 mcg morphine, and 10 mcg fentanyl	No	Intravenous injection of 45.5 mg pheniramine maleate, 20 mg tenoxicam, IV fluid hydration	Full recovery (9 hours)
7[our case]	2021	30	F	Painless bilateral parotid swelling	Elective cesarean section	Spinal anesthesia between L3-L4 intervertebral spaces	8 mL of Marcaine 0.5%, and 10 micrograms of fentanyl	No	Intravenous injection of 100 mg IV hydrocortisone, IV fluid hydration	Full recovery (24 hours)

## Data Availability

All available information regarding this study has been reported in the manuscript. Please contact the corresponding author if you desire further information.
